# Back-table procedure and auto-lung transplantation for locally advanced lung cancer: a case report

**DOI:** 10.1186/s13019-016-0399-x

**Published:** 2016-01-16

**Authors:** Yoko Karube, Masayuki Chida, Morimichi Nishihira, Takashi Inoue, Osamu Araki, Satoru Kobayashi, Tetsu Sado

**Affiliations:** Department of General Thoracic Surgery, Dokkyo Medical University, 880 Kitakobayashi, Mibu, Shimotsuga, Tochigi 321-0293 Japan

**Keywords:** Lung cancer, Auto-transplantation, Bronchoplasty, Angioplasty

## Abstract

**Background:**

To avoid a pneumonectomy and preserve the lung parenchyma, a bronchovascular double-sleeve plasty including an extended sleeve lobectomy is a good choice for locally advanced lung cancer.

**Case presentation:**

We describe a case with lung adenocarcinoma enrolled in our new protocol for *ex situ* auto-lung transplantation following an *en bloc* pneumonectomy and back table procedure for central lung cancer. Following completion of the pneumonectomy, the excised lung was irrigated with a cold extracellular phosphate-buffered solution to protect the lung graft from ischemia-reperfusion injury during preparation of the graft of a right basal segment as a back-table procedure.

**Conclusion:**

Although auto-lung transplantation is a complicated procedure, an *en bloc* pneumonectomy following a back table procedure makes preparation of the graft easy, while simultaneous mediastinal lymph node dissection by another surgeon shortens operation time.

## Background

The risk of perioperative mortality is greater for patients undergoing a pneumonectomy than a sleeve lobectomy. To avoid a pneumonectomy in patients with locally advanced lung cancer, we sometimes perform a bronchovascular double-sleeve plasty including an extended sleeve lobectomy (ESL), an atypical sleeve resection of more than one lobe, to preserve the lung parenchyma [1]. Few reports of *in-situ* auto-lung transplantation with a triple plasty of the bronchus, pulmonary artery, and pulmonary vein for advanced lung cancer have been reported [2–4]. Recently, the advantages of *ex-situ* auto-lung transplantation for central advanced lung cancer have been noted [5, 6]. Here, we present our novel ESL protocol using an en-bloc pneumonectomy followed by a back-table procedure and auto-transplantation of the basal segmental graft.

## Case presentation

A 59-year-old woman was admitted to our hospital with locally advanced lung adenocarcinoma. Chest computed tomography (CT) showed a tumor in the right upper lobe that had invaded the middle lobe and S6 segment, as well as hilar lymphadenopathy (#12u and #11 s) without mediastinal lymph node swelling (cT2aN1M0). An ESL procedure (right upper, middle lobectomies and S6 segmentectomy) that preserves the basal segment with a double-sleeve technique was considered in order to avoid a right pneumonectomy in this patient. Since successful *ex situ* auto-transplantation after a pneumonectomy for locally advanced lung cancer has been reported [5, 6], the Dokkyo Medical University Hospital Ethics Committee approved our protocol (#26030) for an operation including a back-table procedure and auto-lung transplantation after a pneumonectomy for locally advanced lung cancer to examine its merits. Such an auto-lung-transplantation protocol was performed in the present case for curative resection and basal-segment preservation. Consent to participate and to publish this study was obtained from the patient before surgery.

**Fig. 1:**
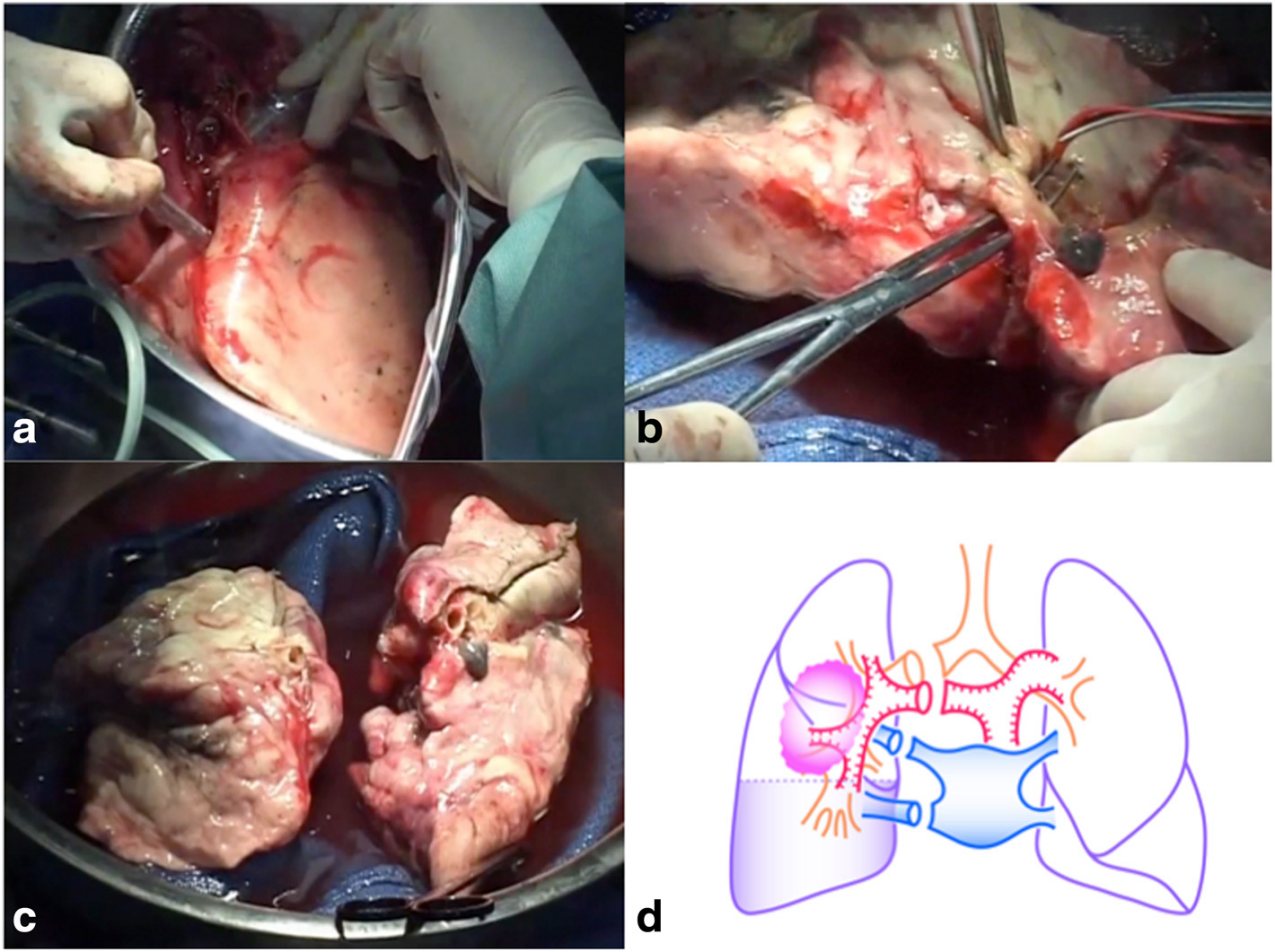
**a** Excised lung irrigated with a cold lung preservation solution on the back table. **b** Division of basal segment graft. **c** Divided graft. **d** Schema of the pneumonectomy and *ex situ* resection

We initially performed a 5^th^ intercostal posterolateral thoracotomy. After administration of intravenous heparin and prostaglandin E1, the pulmonary artery and vein were clamped, and the right pneumonectomy was completed. For a venous anastomosis between the lower pulmonary vein of the graft and upper pulmonary vein of the body, the pulmonary veins were dissected as distal as possible for the upper pulmonary vein and proximal as possible as for the lower pulmonary vein (Fig. 1). Following completion of the pneumonectomy, the excised lung was irrigated with a cold low-potassium phosphate-buffered dextran glucose solution (EP-TU solution; Cell Science & Technology Institute, Sendai, Japan) to protect the lung graft from ischemia-reperfusion injury during preparation of the graft of the basal segment as a back-table procedure. At the same time, another surgeon performed a mediastinal lymph node dissection to shorten the operation time. Thereafter, basal segment auto-transplantation was performed in the order of bronchus, pulmonary artery, and pulmonary vein anastomosis. Finally, the basal pulmonary vein of the graft was anastomosed to the upper pulmonary vein orifice. The operation time was 359 min and blood loss was 305 g.

The postoperative course was uneventful and no ischemia-reperfusion injury was observed, and the patient was discharged from the intensive care unit 1 day after the operation. The pathological diagnosis was pT2aN2M0 adenocarcinoma. Following surgery, the patient received adjuvant chemotherapy with carboplatin and nab-paclitaxel. At 9 months after the operation, she had returned to normal life without recurrence of the primary disease.

**Fig. 2:**
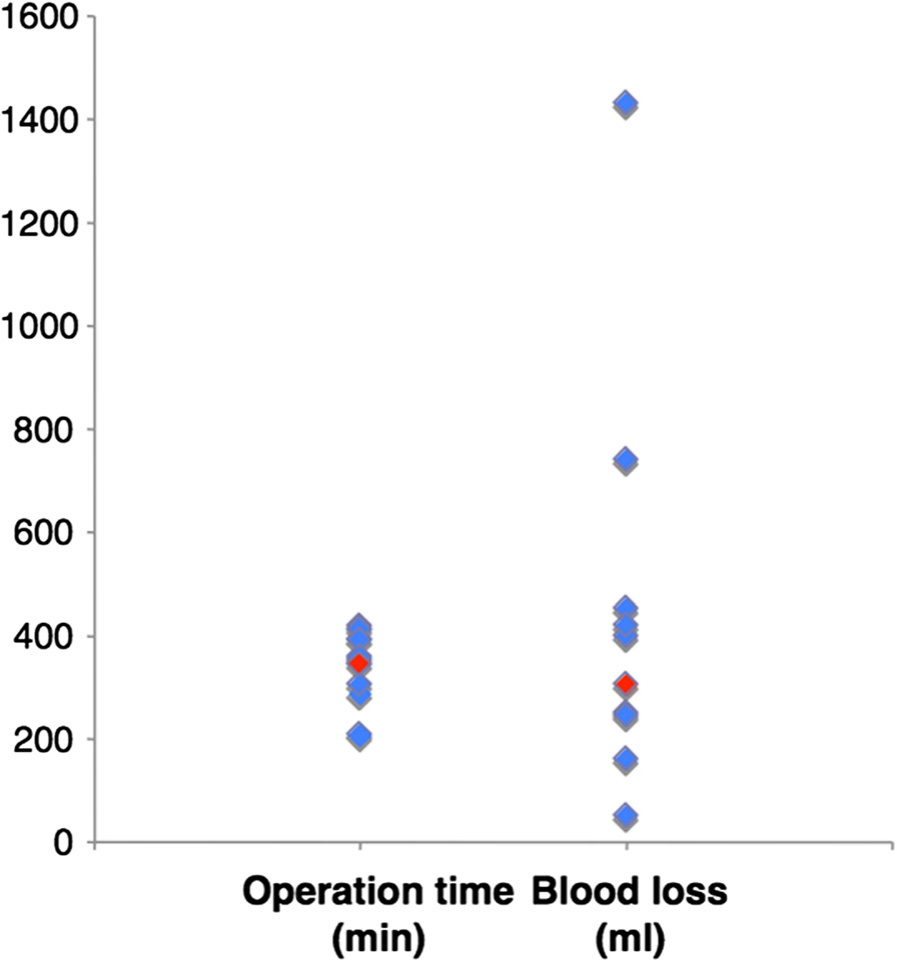
Operation time (*left column*) and blood loss (*right column*) in 13 patients who underwent a basal-segment preserving extended sleeve lobectomy. Red dot indicates the present auto-lung transplantation case

We have performed ESL for 37 patients with locally advanced non-small cell lung cancer at our hospital. Thirty-two of those were considered to have a good risk for undergoing a pneumonectomy, while the others had compromised cardio-pulmonary function. Thirteen underwent a basal-segment preserving ESL, for whom the operation time was 334 ± 75 min and blood loss was 445 ± 394 g. Operation time and blood loss for patients who underwent auto-transplantation preserving the basal segment were similar to those who underwent a usual ESL procedure preserving the basal segment (Fig. 2). Thus, our complicated protocol using auto-transplantation does not increase operation time or blood loss.

## Discussion

The risk of perioperative mortality for patients undergoing a pneumonectomy is considered to be much greater than that for those undergoing a sleeve lobectomy, whereas long-term survival following each is similar. Thus, a pneumonectomy may not be the best choice in all cases, with a sleeve lobectomy or bronchovascular double sleeve lobectomy a reasonable alternative for locally advanced lung cancer.

There are few reports of auto-lung transplantation with an *in situ* triple plasty of the bronchus, pulmonary artery, and pulmonary vein for advanced lung cancer [2–4]. When the inferior pulmonary vein limits tension-free anastomosis of both the pulmonary artery and bronchus, auto-lung transplantation, with the inferior pulmonary vein of the graft implanted into the remaining superior pulmonary vein stump, may be an alternate choice. However, it is important to point out potential problems such as ischemia-reperfusion injury and prolonged operation time associated with this complicated procedure. Reardon et al. found ischemia reperfusion injury in a patient who underwent *in situ* auto-lung transplantation [3]. Jiang et al. reported their experience with 7 cases of *in situ* auto-lung transplantation in which they utilized retrograde-single-lung flushing with heparin solution (500 ml normal saline with 12,500 U heparin at 20 °C). Both of those reports noted the importance of preventing ischemia-reperfusion injury with the lung preservation solution.

Recently, Oto et al. presented a case of auto-lung transplantation for central type advanced lung cancer using a technique of *ex situ* lung irrigation with cold lung preservation solution (low-potassium dextran glucose solution) and *ex situ* division of the graft [5]. Watanabe et al. also reported that in a more complicated case [6]. In reports of lung transplantion, proper lung preservation is recommended for cases in which the warm-ischemic time exceeds 1 h [7], with greater than 2 L of a cold low-potassium phosphate-buffered lung preservation solution recommended for the donor operation. To complete pulmonary vessel irrigation with 2 L of antegrade and retrograde flushing, an *ex situ* back-table procedure following an *en block* pneumonectomy is safer and easier. Furthermore, Oto et al. noted that *ex situ* graft resection on the back table following en bloc pneumonectomy is easier and safer as compared to *in situ* resection [5]. As a result, the operation time may be shortened. In addition, a simultaneous mediastinal lymphadenectomy performed by another surgeon can reduce the time required. In the present case, auto-lung transplantation with use of a back-table procedure did not prolong the operation time as compared to a basal-segment-preserving ESL technique (Fig. 2).

## Conclusion

In conclusion, *en bloc* pneumonectomy followed by a back-table procedure and auto-lung-transplantation for locally advanced lung cancer does not prolong operation time due to both easier lung separation on the back table and simultaneous lymph node dissection by another surgeon. In addition, lung irrigation with cold lung preservation solution prevented ischemia-reperfusion injury in the present case. We are continuing to examine our novel protocol to determine its suitability as a surgical option.
